# A Lower-Energy Pathway for the Creation of Multifunctional Silicon Suboxide Films

**DOI:** 10.3390/ma18050962

**Published:** 2025-02-21

**Authors:** Rita de Cássia Rangel, Rafael Parra Ribeiro, Maria Eliziane Pires de Souza, Danielle Cristina Fernandes da Silva Spigarollo, Gelson Biscaia de Souza, Elidiane Cipriano Rangel

**Affiliations:** 1 Technological Plasma Laboratory, Science and Technology Institute, São Paulo State University (UNESP), Av. Três de Março, 511, Sorocaba 18087-180, SP, Brazil; rita.rangel@unesp.br (R.d.C.R.); rafael-parra.ribeiro@unesp.br (R.P.R.); danielle.spigarollo@unesp.br (D.C.F.d.S.S.); 2 Department of Mechanical Engineering, Federal University of Maranhão (UFMA), Avenida dos Portugueses 1966, Vila Bacanga, São Luís 65080-805, MA, Brazil; maria.eliziane@ufma.br; 3 Laboratory of Mechanical Properties and Surfaces, State University of Ponta Grossa (UEPG), Av. General Carlos Cavalcanti, 4748, Ponta Grossa 84030-900, PR, Brazil; gelsonbs@uepg.br

**Keywords:** SiO_x_, HMDSO, plasma deposition, crosslinking, corrosion resistance, mechanical properties

## Abstract

The possibility of inducing structural crosslinking and densification of plasma-deposited SiO_x_ networks by controlling low-energy reaction mechanisms was investigated. For this, films were deposited for 300 s from HMDSO (2%), O_2_ (86%) and Ar (12%) mixtures at a working pressure of 15.7 Pa. A radiofrequency signal was used to excite the plasma in a configuration so as to not deliberately induce ion bombardment of the growing layers. The plasma excitation power was varied (100 to 300 W) to promote changes in the deposition mechanisms, which were investigated from deposition rate and layer thickness, chemical structure, elemental composition, topography, roughness, hardness, elastic modulus, corrosion potential, corrosion current density and porosity of the films. Under the experimental conditions studied, inorganic SiO_x_ thin films (x = 1.8–1.9) with a low carbon content were deposited. The increase in the applied power during the deposition process reduced the number of silanol groups in the coatings, due to dangling bonds recombination by structural crosslinks, which avoided hydroxyl incorporation and silanol formation. As a consequence, the structure became harder, more compact and corrosion resistant.

## 1. Introduction

Silicon oxide films have found practical applications as protective anti-scratch coatings for ophthalmic lenses [1,2], barrier-type films for food and pharmaceutical packaging [3], coatings for biocompatible materials [4], dielectric layers in microelectronics [5,6] and coatings for metal protection. This wide variety of applications is ascribed to the films’ structural flexibility, enabling the combination of a series of required properties in different applications. Another convenient aspect is the variety of practical approaches available for their preparation, including electrodeposition [7], sol–gel deposition [8], dip coating [9], atomic layer deposition [10] and plasma-enhanced chemical vapor deposition (PECVD) [11]. Of these, the PECVD of organosilicon compounds emerges as a versatile approach, owing to its simplicity, low cost, low consumption of reagents and electrical energy, and the generation of little waste product. These attractive characteristics have stimulated investigations into the use of PECVD of organosilanes [12,13,14,15,16] for the production of SiO_x_ films.

Among the organosilicon compounds, tetraethoxysilane (TEOS) and hexamethyldisiloxane (HMDSO) are the most common precursors for plasma deposition of SiO_x_ films, with their deposition mechanisms having already been extensively investigated [17,18,19,20,21,22,23]. These compounds behave similarly, but HMDSO is more easily fed to the plasma because of its greater volatility (boiling point: about 100 °C for HMDSO and about 170 °C for TEOS).

In atmospheres containing hexamethyldisiloxane (HMDSO, [CH_3_]_3_SiOSi[CH_3_]_3_) [2,3,4,5,6,7], the PECVD process may also provide organosilicon, silicon oxicarbides or a combination of these by varying the deposition conditions [1,2,3,4,5,6,18,19,20,24]. The production of inorganic SiO_x_ groups in plasmas fed with HMDSO and oxygen increases with the addition of argon to the gas mixture [25], with an increase in plasma excitation power [11,26] with pressure [27]. Another possibility is the addition of an oxidant, frequently molecular oxygen, to switch from an organosilicon to an inorganic SiO_x_ coating, by decreasing the organosilicon-to-oxygen ratio in the plasma feed [17,18,28,29]. Nitrogen-containing precursors mixed with oxygen can also be utilized for SiO_x_ deposition [25], leading to silicon nitride-like thin films [26,27]. Besides the feed composition, other parameters also play important roles in determining the final film composition, structure, hardness and porosity: power density, excitation frequency, surface temperature and ion bombardment [19,24,29].

There is consensus in the literature that films with low organic proportions are more efficient for corrosion protection [1,3,11] than organosilicon because of their greater density and chemical inertness. However, factors such as the adhesion of silica to the substrates (metal or polymer), mechanical stress, and especially the defects generated in the deposition process or by substrate finishing, affect the efficiency and physical stability of the layer and, therefore, its practical application.

Petit-Etienne et al. [27] determined the porosity of silica films deposited from HMDSO plasma and found that the concentration of these defects decreases with greater ion bombardment intensity. Therefore, although the deposition occurs at the atomic and molecular scale, defects are incorporated and their minimization is of great relevance to the barrier properties of the films.

In the work of Milella et al. [30], an increase in SiO_x_ film density was shown to be caused by post-deposition ion bombardment in an argon and oxygen plasma [30]. The densification and the topographical smoothening were dependent on the treatment time and voltage applied to induce ion bombardment. In another work by the same authors [2], it was shown that the bombardment of the material surface by low-energy ions can lead to the breaking of chemical bonds, diffusion and heating; factors that contribute to film densification and to a reduction in structural disorder. Excessive bombardment energies, however, may damage the structure and cause the internal tension of the layer to grow. These results were confirmed by the work of Lefèvre et al. [31], who used molecular dynamic simulations to show that ionic bombardment during SiO_x_ film growth promotes structural changes, which affect the density and internal stress of the films. The films deposited in the absence of ion bombardment presented high tensile stress and, consequently, reduced adhesion to the substrates and resistance to cracking. The low deposition temperature was indicated as the factor responsible for this result.

Blanchard et al. [32] proposed a method for the densification of plasma-deposited organosilicon films via the control of plasma excitation power and the self-bias of the driven sample holder. In this work, the role of excitation power in homogeneous (gas phase energetics) and heterogeneous (surface ion bombardment) plasma reactions was discussed. Densification is proposed to be not only related to ion bombardment during film deposition, but also to the plasma activation state, characterized in terms of the energy delivered per molecule of the plasma phase.

Therefore, considering the importance of densification for protective layers, and that the studies in the literature report the densification of silica-based material in association with ion bombardment or heating during or after the deposition process, the goal of the present work is to investigate the possibility of increasing the density of silica-based films by tailoring homogenous and heterogeneous low-energy reactions. The system’s electrical configuration was specifically designed to prevent ion bombardment of the growing layer. Samples were attached to the floating potential electrode, restricting the deposition process to low-energy chemical reactions. The power of the excitation signal was selected as the key experimental variable, since it directly influences the energy of the plasma species. The power range employed in this study (100–300 W) was delimited in order to modify the deposition kinetics while preserving the silicon oxide nature of the films. Although several reports have already been published on the effect of plasma excitation parameters on the properties of silicon-based films, the role of plasma phase reactions and plasma surface energetic interactions in structure densification are not fully understood [32,33].

## 2. Materials and Methods

Films were deposited in a parallel-plate reactor consisting of a stainless-steel cylindrical chamber, pumped by a turbomolecular-rotary pumping system. The injection of compounds into the reactor was regulated by mass flow controllers and a vapor source controller (MKS Instruments, Inc., Andover, MA, USA). The internal pressure was monitored by a baratron gauge and controlled by an automatic throttle valve. The lower electrode, at floating potential, was used as the sample holder. When at floating potential, the electrode does not attract charged plasma species, thereby preventing ion or electron bombardment, as necessary for testing the stated hypothesis.

Ultrasonically cleaned substrates were accommodated in the sample holder and the system pressure was reduced to 1.7 Pa. Film deposition was performed for 300 s in an atmosphere composed of HMDSO (0.21 Pa, 4 sccm), O_2_ (8.11 Pa, 120 sccm) and Ar (1.15 Pa, 45 sccm). The initial pressure of the gasses (9.5 Pa) was then adjusted to 15.7 Pa by reducing the pumping velocity but maintaining the proportions of the gasses. The plasma was generated by applying a radiofrequency signal (13.56 MHz) to the upper electrode, while the reactor walls were grounded. In all the experiments, the deposition time was 300 s. The effects of plasma excitation power, which was varied from 100 to 300 W, on the films were investigated.

The film thickness (h) was determined from the height of a step produced by film deposition while masking part of the Si wafer with a glass plate. The step height was measured in at least seven different regions with the aid of an Alpha-Step 500 KLA Tencor profilometer (Tencor Instruments, Mountain View, CA, USA). The deposition rate (R) was derived from the ratio of the thickness and the deposition time.

The chemical structure of the films deposited onto polished silicon plates was analyzed by infrared reflectance–absorbance spectroscopy (IRRAS) in a Bruker Equinox 55 spectrometer (Bruker Scientific Instruments, Billerica, USA). For each spectrum, 32 scans were performed in the 375–4000 cm^−1^ range and with resolution of 4 cm^−1^. The elemental composition and chemical bonding state of the film surfaces were derived using X-ray photoelectron spectroscopy (XPS). Data were acquired in a PHI 5300 ESCA spectrometer using MgKα radiation and with 0.05 eV of energy resolution. High-resolution spectra (Si 2p) were registered at an electron take-off angle of 45° and after 20 s of surface cleaning using Ar^+^ ion sputtering (1 keV). High-resolution spectra were calibrated in energy by the reference Si 2p peak at 103.4 eV (SiO_2_) and fitted with Gaussian peaks after subtraction of the Shirley-type background. XPS analyses were conducted on the samples deposited onto the silicon plates.

The mechanical and tribological properties of the films were examined, respectively, by nanoindentation and scratching tests in a Hysitron TI 750 UbiTM Nanoindentation system with a performechTM control scanning unit. Indentations were conducted at 15 different positions on each sample, using a diamond Berkovich tip and a partial unload function of 5000 μN. The hardness and elastic modulus were calculated from the load displacement curves according to the Oliver Pharr method [34]. The tip area function was calibrated from the results of indentations on fused quartz before the experimental test.

Topographic images of the surfaces were taken before and after indentations by scanning the same Berkovick tip, under a 2 µN load, over a 5 µm × 5 µm area. For the scratching experiments, a ramp function of 200 µN of maximum load was applied to the tip while displacing it by 10 µm. To evaluate the surface damage, new AFM topographic profiles (10 µm × 10 µm) were taken after the scratching procedure. All of the above-mentioned mechanical and tribological tests were performed on samples prepared in silicon substrates. However, for inspecting the behavior of the films under higher loads and in different substrates, scratching experiments were repeated on samples prepared on carbon steel substrates. For this, a ramp function of 30 mN of maximum load was applied to the tip while displacing it by 80 µm with 2 μm/s of speed. To evaluate the surface damage, optical images were taken using the microscope coupled to the nanoindenter system.

The corrosion resistance of the as-received film and the carbon steel-containing film (NBR 5915) plates, in NaCl solution (3.5%), was derived from potentiodynamic polarization measurements. The analysis was carried out in a conventional electrochemical cell with three electrodes. Carbon steel samples were used as the working electrodes while the counter and reference electrodes were platinum/rhodium and silver plates, respectively. The current density was recorded while changing the potential from the cathodic region (−0.25 V) to the anodic one (0.25 V), at a rate of 0.17 mV/s. The linear regions of the anodic and cathodic portions were extrapolated to derive the corrosion potential, E_corr_, and the current density, I_corr_, as proposed by Zhang et al. [35].

## 3. Results

### 3.1. Thickness and Deposition Rate

**Figure 1:**
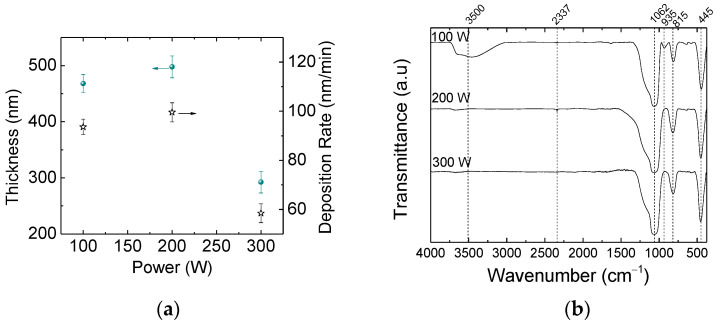
(**a**) Thickness and deposition rate of films as a function of plasma excitation power. (**b**) Infrared spectra of films deposited in plasmas of different powers.

The thickness, h, and deposition rate, R, of the films are shown in Figure 1a as a function of the plasma excitation power, P. There is a slight variation in h as P is increased from 100 to 200 W, but a steep drop (~50%) appears at the highest *p* value (300 W). As deposition time was kept constant (300 s) in all the experiments, the deposition rate follows exactly the same trend as h, decreasing from 95 to 59 nm/min (38%) as P is increased from 100 to 300 W.

Mahajan et al. [36] also reported a reduction in deposition rate with increasing deposition power. The authors ascribed this trend to the back etching of species during deposition. On the other hand, Vendemiatti et al. [37] reported an increase in deposition rate as the proportion of oxygen in O_2_/Ar/HMDSO mixtures increased from 0 to 30% (while Ar was decreased from 30 to 0%). However, at the high HMDSO proportion (70%) used in their work, the oxygen content was not sufficient to fully fragment oxide groups, but only to enhance the concentration of polymerizable (CH_3_)_3_-Si-O-, (CH_3_)_3_-Si-O-Si-O-(CH_3_)_2_, Si-(CH_3_)_3_ and CH_3_ species in the plasma phase, thus accelerating the deposition rate.

To understand the results of Figure 1a, it should be taken into account that the plasma activity [38], defined as the product of the average electron energy and its density, increases with P, making the activation process more effective. Thus, the falls observed in the curves of Figure 1a are ascribed to the intense dissociation of O_2_ and HMDSO molecules, caused by an elevation in plasma activity along with the action of oxidative reactions. Together, they promote growth in the content of highly fragmented/oxidized species (C, H, CO, CO_2_, OH, H_2_O, etc.) from HMDSO, which are volatile and do not contribute to film growth. Thus, although the precursor fragmentation rate increases with power, the overall availability of film-forming species decreases, explaining the reduced deposition rates observed at the highest power.

Besides increasing the average energy and density of the activated species, increased power also increases its reactivity, since a greater variety of fragments are generated. It was demonstrated, for instance, that the concentration of oxygen radicals increases with P in pure O_2_ plasmas, especially in high-pressure processes, such as those employed in the present work (16 Pa) [24]. The overall availability of film-forming species is reduced by oxidation, while the removal of newly deposited groups of the film by etching is increased, with both effects contributing to the diminishing trends shown by the curves of Figure 1a.

### 3.2. Chemical Structure and Elemental Composition

The infrared spectra of the films investigated here are shown in Figure 1b. Table 1 summarizes the wavenumbers of the main absorption bands found in the spectra and their respective assignments. The most prominent band, lying at 1062 cm^−1^, is associated with the Si-O stretching vibration of SiO_x_ groups. When this band is centered between 1075 and 1080 cm^−1^, a SiO_2_ stoichiometric structure is expected [39]. When shifted to lower wavenumbers, however, as in the spectra of Figure 1b, a SiO_x_ non-stoichiometric network is indicated. The shift in the vibrational frequency downwards derives from the replacement of Si-O by Si-Si bonds, thus revealing further chain connections and a denser silicon-based material due to crosslinkings [40]. The full width at half maximum (FWHM) of this contribution is also dependent on the structural organization. In the stoichiometric SiO_2_ structure, the FWHM of the band is around 80 cm^−1^, while wider peaks, such as that observed at 224 cm^−1^, are found in SiO_x_ crosslinked networks [40]. The bands at 815 and in 445 cm^−1^, detected in all the spectra, are associated, respectively, with the rocking and bending modes of Si-O.

The spectrum of the film deposited at the lowest power, 100 W, presents two other bands at 935 and 3500 cm^−1^, which are attributed to OH vibrations in Si-OH. The abstraction of methyl groups from the methylsilyl functional, in the solid or plasma phase, traps free radicals in the film structure. The incomplete consumption of dangling bonds by chain crosslinking keeps residual radicals active in the film. The permeation of oxygen and water vapor through the structure as the sample is exposed to air eventually saturates the bonds by incorporation of hydroxyl groups. The incorporation may also occur by heterogeneous low-energy reactions taking place during film deposition. Besides preventing the establishment of Si-Si connections, SiOH groups act as reactive points where degradation reactions may be initiated when in contact with water or its vapor [39]. Therefore, the presence of the SiOH functionals suggests a more reactive and less crosslinked structure, which are negative points in relation to mechanical and chemical resistance as well as structural permeability. Finally, the presence of free-OH molecules in the structure is revealed by the increase in the low-intensity band at around 3650–3690 cm^−1^ [41].

It should be taken into account here that a diversity of fragments can be generated from the HMDSO molecule, depending on the plasma activity [38]. When deposition is conducted in the low-fragmentation regime, that is, using low power or high flow ratios, the oligomerization of high-molecular-weight fragments gives rise to species much larger than the original precursor molecule [42]. The deposition of such huge, organic-rich molecules generates an organosilicon structure very similar to that of conventional polydimethylsiloxane (PDMS) or silicone. In this case, the presence of methylsilyl (Si(CH_3_)), dimethylsilyl (Si(CH_3_)_2_) and trimethylsilyl (Si(CH_3_)_3_) groups connected to the Si-O-Si backbone [43] is readily detected in the infrared spectrum of the material by an absorption band lying at 1260 cm^−1^. Furthermore, depending on the degree of activation, a crosslinked PDMS-like structure is generated, such as the one proposed by Blanchard et al. [32] and Rangel et al. [44].

On the other hand, when high excitation powers [45] or elevated proportions of oxygen [43,46], or both, are used, there is severe fragmentation of the organosilicon precursor. In the oxygen-rich atmosphere, the fragments can be further dissociated and oxidized, generating CO, H_2_O and OH volatile groups [43]. As these low-molecular-weight species have low sticking probabilities [45,47] they do not effectively contribute to film formation, being eventually removed from the deposition chamber. Heterogeneous plasma surface reactions also contribute to the abstraction of organics from the deposited material [43], generating extra pendant bonds. The deposition rate is reduced as a consequence. When such mechanisms predominate, there is the formation of a SiO_2_-rich structure with organics appearing just as contaminants. This regime, proposed as an explanation for the results of Figure 1b, is based on a deposition mechanism involving low-energy deposition by ion bombardment, which is consistent with a sample holder at floating potential.

The films’ elemental composition and chemical bonding state were analyzed by XPS. The detected elements were carbon (C), oxygen (O), and silicon (Si) and their atomic proportions are presented in Table 2, together with their O/Si atomic ratios. Only traces of C (≤1%) were detected, while O (65%) and Si (34%) represented the main elements of the film surface. Furthermore, no significant changes in these concentrations were detected as the applied power was varied.

In a study by Wavhal et al. [43] changes in the concentration of C and O were observed as the excitation power was changed from 25 to 125 W; however, a stabilization was detected for the highest excitation powers, in good agreement with the results presented here: the low carbon content and the O/Si ratio around 1.8–1.9 characterize a SiO_2_-like structure in which an ideal ratio of 2.0 is expected. Therefore, the excess of Si with respect to O corroborates the idea of the replacement of Si-O by Si-Si bonds and structural crosslinking enhancement.

**Figure 2:**
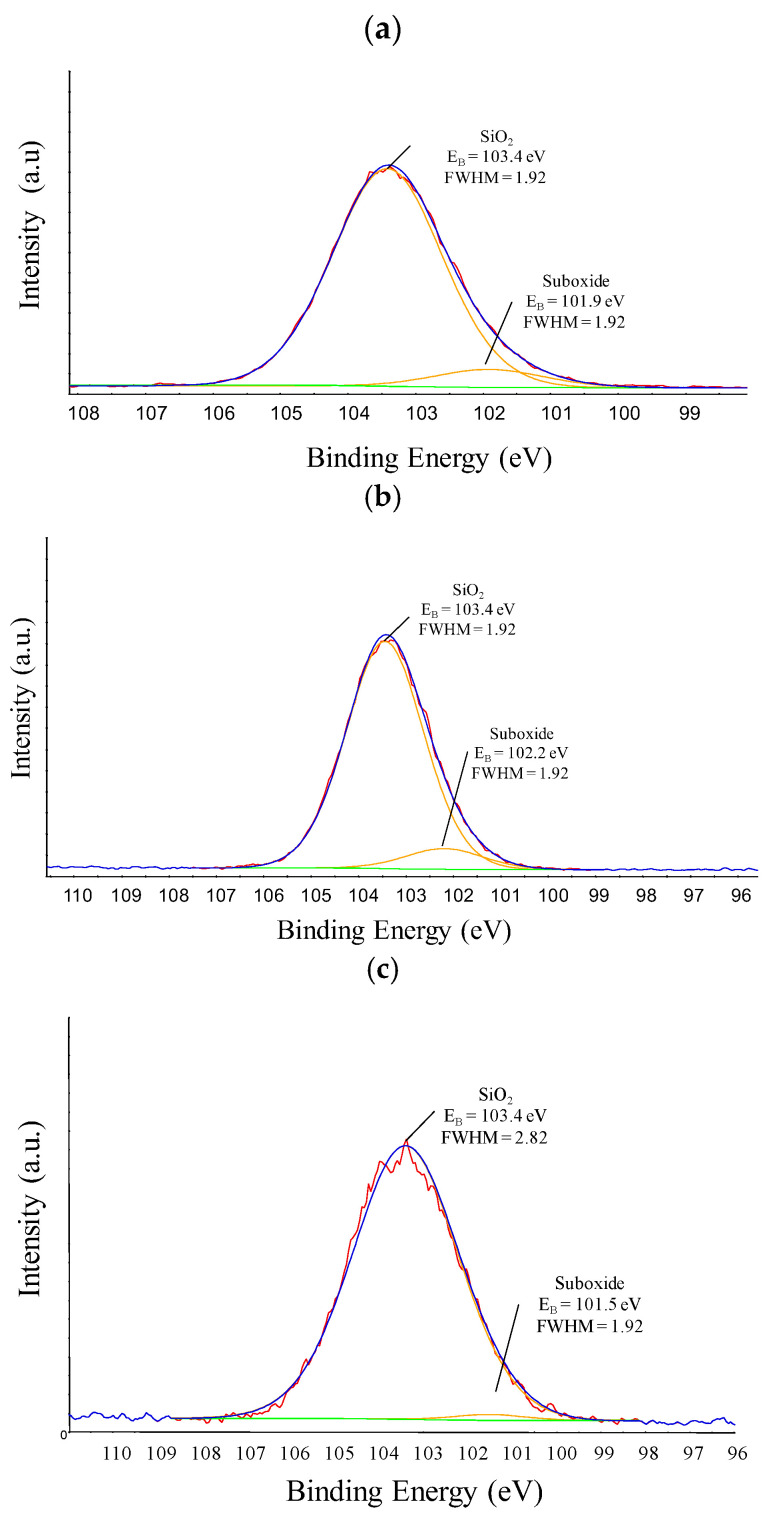
Si 2p high-resolution XPS peak of films deposited in plasmas of different powers: (**a**) 100 W, (**b**) 200 W, (**c**) 300 W. The red line represents the experimental data, the blue curve depicts the simulated spectrum, while the orange and green lines correspond to the individual components of the simulated peak.

The high-resolution Si 2p spectra obtained from the films studied here are presented in Figure 2. The adjustment of the peaks was conducted considering silicon surrounded by four Si(-O_4_) (103.4 eV) atoms, two Si(-O_2_) (102.0 eV) atoms and one oxygen (101.5 eV) atom. These environments for Si are consistent with those used in previous reports within 0.1 eV of tolerance [32,43,48]. The proportion of each contribution to the total area of the peak was determined and is presented in Table 3. As can be noticed, the coating is predominantly composed of tetrahedral silicon oxide, with the suboxide network appearing at low proportions (<10%).

**Figure 3:**
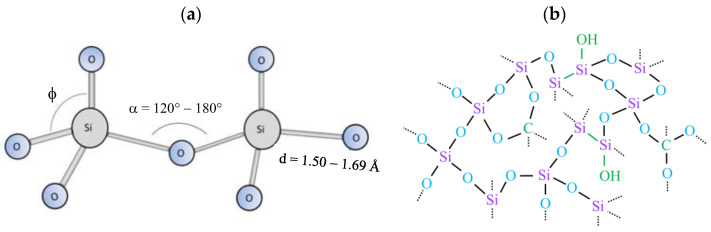
A schematic illustration of the (**a**) silicon dioxide group in the tetrahedral configuration and (**b**) silicon dioxide amorphous structure proposed in the present work. The green highlights detail the silanol moieties and carbon contaminations, as well as the Si-Si crosslinks. The dashed lines indicate binding points with the substrate or adjacent chains.

The rise in the SiO_4_ component, which is schematically represented in Figure 3a, reflects high fragmentation imposed to the organosilicon molecule [43]. In these three-dimensionally connected networks, C and hydroxyl moieties are linked to suboxide groups, as depicted in the illustration of Figure 3b. The high proportion of SiO_4_ in the sample prepared at 300 W thus reveals a high-quality oxide structure, obtained without biasing the substrate holder, as is normally reported in the literature [24,27,49], confirming the hypothesis that ion bombardment is not the only mechanism responsible for this.

The results of this section reveal the formation of a non-stoichiometric three-dimensionally connected SiO_x_ structure (100 W), with the degree of crosslinking (Si-Si) rising with increasing deposition power up to 200 W, and its eventual evolution into an almost complete tetrahedral network (300 W). Organic contamination was low compared to that found in other works [43,48]. Variation in excitation power, in the range used here, did not change the nature of the film, but changed its spatial organization, crosslinking degree and preventing organic and SiOH incorporation. The schematic representation of such a structure is presented in Figure 3b, where the green highlights detail the silanol moieties, carbon contaminations and Si-Si crosslinks.

### 3.3. Surface Topography and Roughness

**Figure 4:**
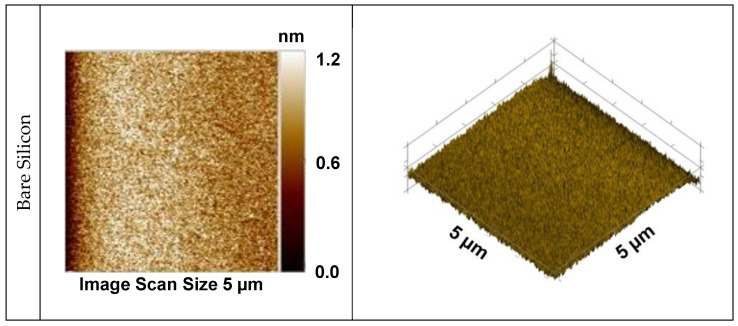

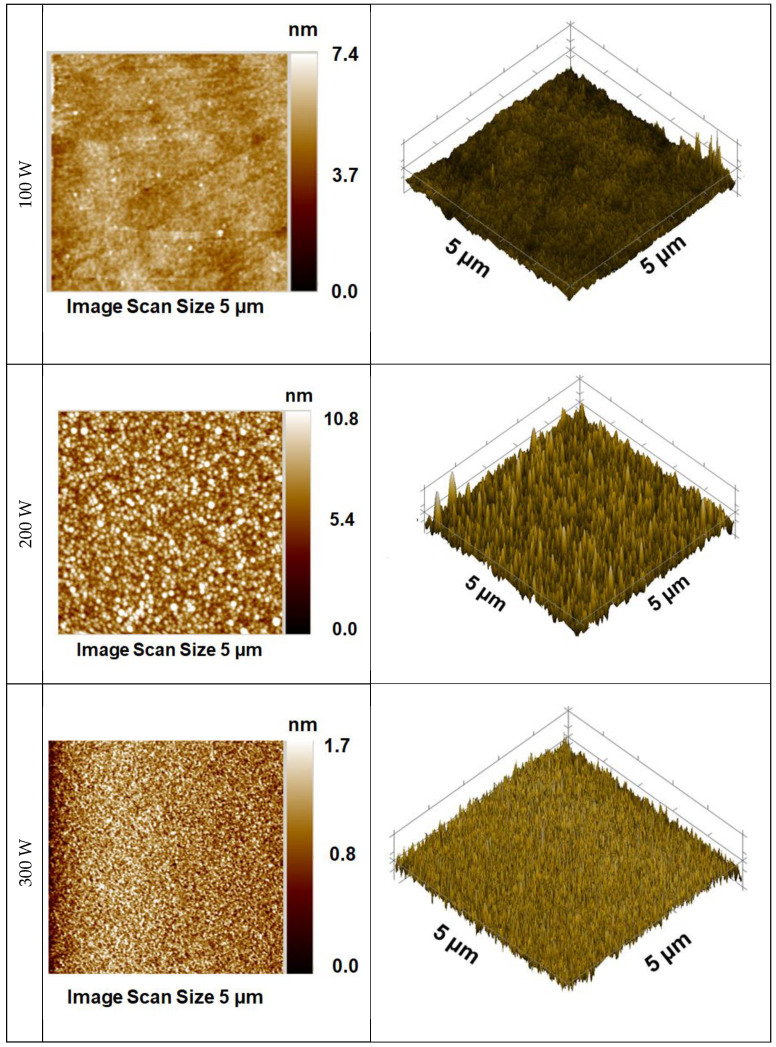
Two- (**left**) and three- (**right**) dimensional surface topographic images (5 μm × 5 μm) of the bare silicon substrate and of the silicon-containing films deposited in plasma of different powers.

The surface microstructure of a film plays a decisive role in its protective performance. For instance, surface roughness is known to influence the anodic dissolution of metals [50]. Additionally, hardness values obtained by nanoindentation are inherently dependent on surface roughness [51]. Owing to this, the surface microstructure of the films was also examined. Figure 4 shows 2D and 3D representative topographic images of the bare silicon substrate and of the silicon-containing SiO_x_ films. The polished silicon has a smooth and flat surface, as observed from the reduced value of the scale of its profile. In general, the surface is uniformly coated by the film, with no evidence of cracks, detachment or uncoated areas, but with a clear change in the surface morphology after plasma exposure, suggesting the presence of the film.

For the film grown at the lowest applied power (100 W), the surface presents an apparently uniform matrix with white spots sparsely spread upon it. Deposition seems to occur uniformly on the substrate surface, with no preferential points for film growth. A similar topography was reported by Zanini et al. [6], when a SiO_x_ coating was prepared from an HMDSO/O_2_ mixture at moderate plasma pressures.

As P is enhanced (100–200 W), the concentration and size of the regular rounded structures increase. The regular matrix is replaced by posts with heights of several nanometers, giving rise to a fakir-like surface, which is typically found in glassy SiO_2_ films [52].

Eventually, topographical smoothing is detected (300 W), with a sudden reduction in the diameter and height of the spikes. As the topographic profile of this sample resembled that of the silicon wafer, new inspections were conducted to verify whether the film was still adhered. The film was readily detected by the unaided eye and infrared spectroscopy inspections, revealing the creation of a fine-grained packed structure.

Spherical particulates on the surface of SiO_x_ films have also been reported in previous works [53,54]. The concentration and size of nano-spherical agglomerates in silicon oxide coatings was observed to strongly depend on the proportion of O_2_ admitted to the plasma environment in the work of Huan et al. [55]. According to the same study, structures composed of larger aggregates of particles give rise to a greater number of pores, which allow gas and moisture permeation, yielding coatings with poor barrier properties.

To understand these results, it should be taken into account that the reduction in deposition rate, demonstrated in the graph of Figure 1a, reveals a modification in the deposition kinetics. The first elevation of power from 100 to 200 W, without changing the deposition rate and thickness, favors the plasma polymerization process, and explains the enlargement of globular structures in the film. The elimination of organics from the precursor molecules with further increasing of P to 300 W reduces the size and availability of film-forming species. A reduction in the rate at which they arrive at the surface gives extra time for re-arrangements to occur. The incorporation of defects (voids) thus decreases with both a reduction in the size of the depositing fragments and their arrival rate. Therefore, the structural evolution observed here can be attributed to the heightened fragmentation process induced by low-energy reactions, which impact both the concentration and size of the agglomerates in two distinct ways.

**Figure 5:**
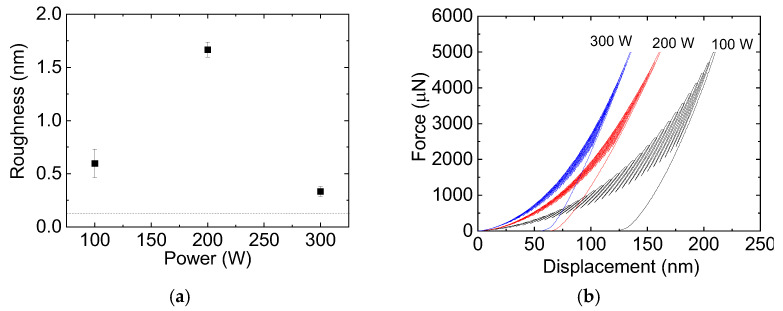
(**a**) The root mean square roughness of the samples as a function of the plasma excitation power. The dotted line in the graph represents the root mean square roughness values for the bare silicon substrate. (**b**) The load–displacement curves obtained in the nanoindentation experiments on films deposited in plasmas of different powers. A partial unload charge function of 5000 μN was used in the tests.

The root mean square (RMS) roughness of the samples, derived from the total image area (5 μm × 5 μm), is shown in Figure 5a as a function of P. The roughness values found for the silicon substrates, represented by the dotted line in the graph, ranged from 0.20 to 0.23 nm. These values are consistent with those found in the literature ranging between 0.01 and 0.57 nm [56,57]. The system roughness increased after film deposition, independently of the condition which was employed, with a steady rise appearing with an increase in power from 100 to 200 W. This result is ascribed to the enhancement in the pillar heights. Since the film thickness was not significantly changed with this power modification, the extra energy provided by the greater power process was used to change the deposition mechanisms, as discussed before. Consistently with the topographic profiles, roughness fell with an increase in deposition power beyond 200 W.

Despite being more compact and inert than its organosilicon (SiO_x_C_y_H_z_) counterparts, silica films present defects generated during the deposition process or by the substrate mold effect. Together with these, the internal stress, incorporated in the coating during its deposition, accounts for defect creation. In the work of Petit-Etienne et al. [27], for instance, a reduction in the porosity of silica films was observed when ion bombardment was induced during deposition. Milella et al. [30] demonstrated densification and smoothing of SiO_x_ films deposited by sputtering when post-deposition ion bombardment treatments were conducted, affecting the optical and the electrical properties of the system. In the present work, the changes in the surface topography are attributed mainly to alterations in the plasma kinetic with P, since ion bombardment is not very effective when the substrate is at floating potential.

Therefore, even though the power of the plasma excitation signal barely affected the chemical composition, it strongly influenced the surface topography. With increasing P, there was a transition from a rather uniform matrix to a fine-grained smooth SiO_x_ structure. This modification aligns closely with the increase in the content of SiO_4_ tetrahedral oxide observed in the structure as the power increased (Table 3).

### 3.4. Mechanical and Tribological Properties

Figure 5b shows the representative nanoindentation load–displacement curves for the SiO_x_ coatings deposited on silicon, which were obtained using a partial unload function (5000 µN). In all cases, the loading and unloading segments are smooth, regular and free of discontinuities, indicating that the film adhered to the substrate which did not crack under stress [58]. The maximum penetration depth can be observed to decrease with increasing deposition power, indicating a greater tip penetration resistance. In addition, it can be noticed that the area under the curve for the film prepared with the lowest power (100 W) differs significantly from the others, indicating a higher plastic deformation.

**Figure 6:**
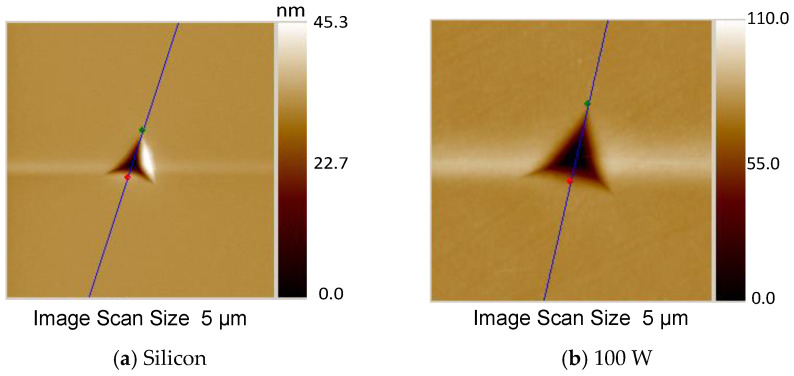

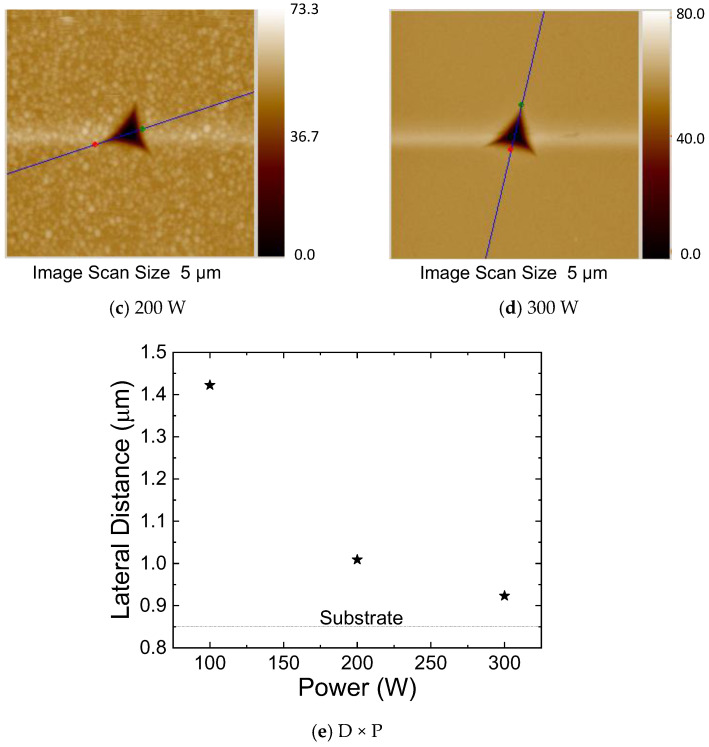
Atomic force microscopy profiles (**a**) of the bare silicon and (**b**–**d**) of the samples submitted to depositing plasmas of different powers, and after indentation with a multiple load function with 5000 µN of maximum force. The inspected area has an extension of 5 μm × 5 μm. In (**e**), the lateral distance along the indention profile, as a function of P, is presented.

Figure 6a–d show the 2D topographic profiles of the surfaces taken after the nanoindentation procedure. The corresponding result for the bare Si substrate is also included. All the indented areas reveal plastic deformation without any cracks around the corners, the region where the highest stress is concentrated. The absence of cracks indicates a material with fracture toughness and reliable results for elastic modulus and hardness [24,59].

From the topographic profiles of Figure 6, the lateral distance of the impress, D, was measured along the indent profile, as defined in the work of Tekaya et al. [60]. The results are presented in Figure 6e as a function of P. A decrease in D with increasing P can be observed, corroborating the idea of an elevation in resistance to plastic deformation [60].

**Figure 7:**
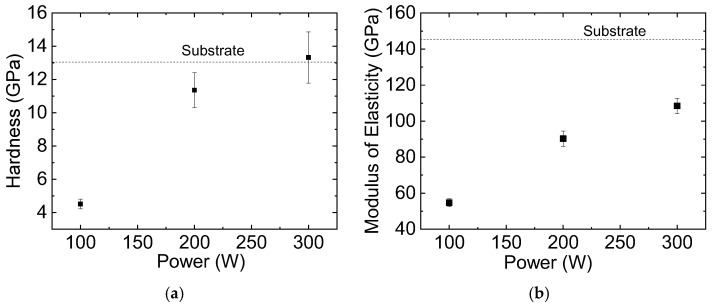
(**a**) Hardness and (**b**) modulus of elasticity of the films as a function of plasma excitation power. Dashed lines in both graphs represent, respectively, hardness and elastic modulus of bare silicon substrate.

The hardness of the samples, H, determined at depths representing 15% of the total layer thickness [61,62,63], is depicted in Figure 7a as a function of the plasma excitation power. The film deposited using the lowest P (100 W) presents hardness values that are close to those reported for SiO_x_ systems [58,64]. Hardness increases threefold (4.5 to 13.3 GPa) as P is changed from 100 to 300 W, overcoming the values reported for silica-like films [61,65,66,67], and approaching those measured for crosslinked SiO_x_ networks [24] and fused silica [68].

Figure 7b shows the effect of the depositing power on the reduced elastic modulus, E_r_, of the samples, which was determined in the substrate-interference-free region [62,63,69]. Similarly to the observations in the hardness results, there is a progressive increase in E_r_ with increasing P. Then, the upward shift in both of the curves in Figure 7 suggests a structural densification when P is increased.

The mechanical properties of the films prepared at 300 W are summarized in Table 4, along with the values reported in the literature for various SiO_x_ structures. In films deposited using low-pressure plasmas, deposition power plays a critical role in determining H and E_r_. Despite variations in other deposition parameters across different studies, H and E_r_ generally increase with power under low-pressure PECVD depositions. The values obtained in this study at 300 W are consistent with those reported for silica films and thermal oxides [67,68], but are significantly higher than those observed for silicon oxides deposited via CVD [70]. In contrast, atmospheric plasma (CAP) deposition produces hardness and reduced elastic modulus values approximately an order of magnitude lower [71].

**Figure 8:**
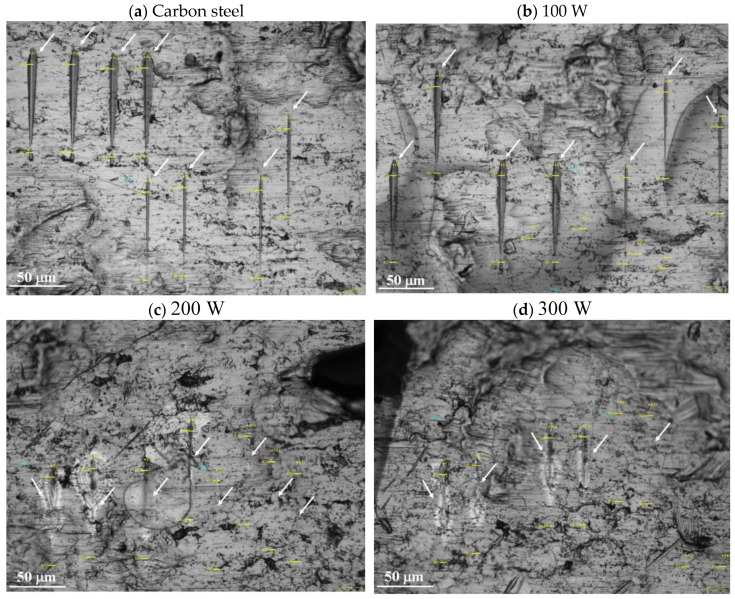
Optical microscopy images of the surfaces which were submitted to the scratching tests: (**a**) the bare carbon steel substrate and carbon steel exposed to depositing plasmas of (**b**) 100 W, (**c**) 200 W and (**d**) 300 W, after the scratching test using a ramp function with 30 mN of maximum load and 80 µm of extension. The arrows in the images indicate the scratched regions.

Figure 8 shows optical microscopy images of the surfaces which were submitted to the scratching tests, using a ramp function with 30 mN of maximum load and 80 µm of extension. Carbon steel plates were used as substrates, which is the reason why the correspondent result for the bare carbon steel is also included.

In all cases, no cracking or peeling of the portion nearby the scratched region can be observed, indicating the formation of a rigid structure well adhered to the substrate [18]. Nevertheless, the deposition power can be observed to affect the width and depth of the tracks produced in the different samples: shorter, shallower and thinner risks are observed in the sample prepared with highest power (300 W), revealing an increment in the scratching resistance with increasing P. This is a notable result, since it would directly impact on the durability of a device coated with this film.

**Figure 9:**
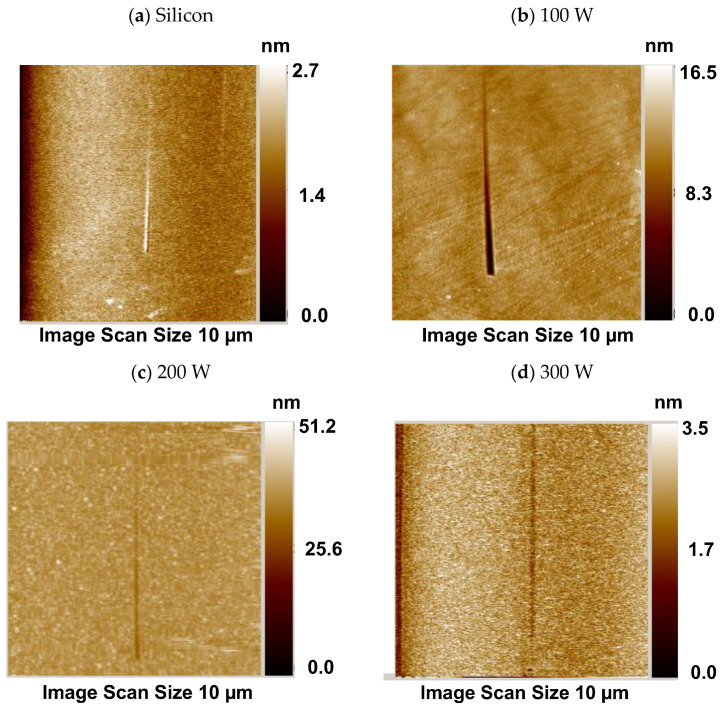
Atomic force microscopy profiles of the (**a**) bare silicon substrate and of the silicon exposed to depositing plasmas of (**b**) 100 W, (**c**) 200 W and (**d**) 300 W, after the scratching test using a normal force of 200 μN. The inspected profiles have an extension of 10 μm × 10 μm.

Scratching tests were also performed on the samples prepared on silicon substrates, using smaller loads and scans than the previous ones. Figure 9 shows 10 µm × 10 µm topographic profiles of the surfaces submitted to the scratching tests using a ramp function with 200 µN and 10 µm of extension. The corresponding result for the bare Si substrate is also included. Consistently, the same trends observed for the samples prepared on carbon steel are also detected when silicon is used as a substrate at a lower scale.

The improvement in the mechanical and tribological properties with increasing power is consistent with the reduction in the silanol number and with the consequent elevation of the crosslinking degree by means of Si-Si connections. The general best performance of the sample prepared in the highest-power plasma (300 W) agrees well with the detection of a predominantly tetrahedral SiO_2_ network for this coating.

### 3.5. Barrier Properties

**Figure 10:**
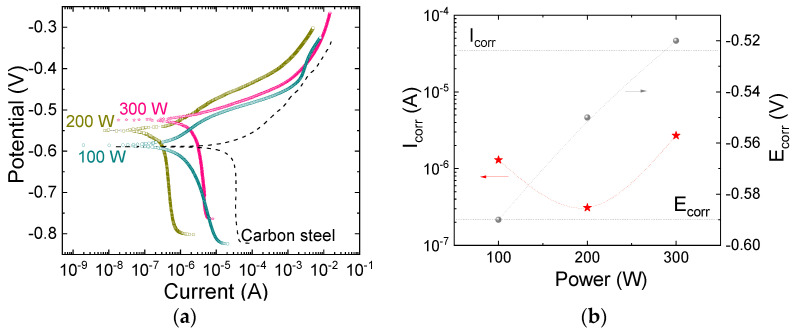
(**a**) Potentiodynamic polarization curves for the bare substrate and for the film-containing samples. (**b**) The corrosion current density and potential as a function of the power employed in the plasma deposition process. The dotted and dashed lines indicate the corresponding properties for the bare carbon steel.

Figure 10a shows the anodic and cathodic portions of the potentiodynamic polarization curves for the bare carbon steel (dotted line) and for the film-containing samples. The cathodic portion of the curves shifts as the carbon steel is coated with the film, but its shape is preserved. This result demonstrates variation in the corrosion rate while the corrosion mechanism is preserved [69]. The anodic portion of the curve also dislocates for the film-containing systems, but its form is progressively changed with varying P, showing that the corrosion proceeds in different ways when the coating is present.

The corrosion current density, I_corr_, and potential, E_corr_, were evaluated from the potentiodynamic curves, and are presented in Figure 10b as a function of P. The corrosion potential for the bare carbon steel (−0.59 V) is observed to shift upwards after film deposition, this increase being dependent on P.

The I_corr_ decreases from 35.00 μA for the bare carbon steel (dotted line) to 0.31 μA for the sample prepared in plasma of 200 W. Even though the samples treated in plasmas of 100 and 200 W presented roughly the same thickness (Figure 1a), the latter resulted in a corrosion current one order of magnitude lower. The better result for this sample correlates well with the depletion of SiOH groups and the consequent production of a crosslinked SiO_x_ network. Besides inhibiting crosslinking, OH groups act as reactive points where degradation may be initiated. The dislocation of the E_corr_ to noble potentials simultaneously with the reduction in I_corr_ corroborates an increase in the degree of crosslinking, in good agreement with previous results [74,75,76].

Despite possessing a higher-quality silicon dioxide structure, the sample prepared at 300 W presents an I_corr_ ten times greater (3.00 µA) than that of the film deposited at 200 W (0.31 µA). To further understand such behavior, the porosity of the films was evaluated from the data of Figure 10 and using the following expression, proposed by Li et al. [77]:

$$Porosity=\frac{R_{ps\left(substrate\right)}}{R_{pc\left(coating-substrate\right)}}\times 10^{-\left|∆E_{corr}\right|/\beta_{a}}$$

In this expression, *R_ps_* is the polarization resistance of the substrate, *R_pc_* is the polarization resistance of the coating, Δ*E_corr_* is the corrosion potential difference between film and substrate and *β_a_* is the anodic Tafel slope for the substrate. The results are presented in Table 5 as a function of P. All the coatings present low values of porosity, with the minimum value occurring when deposition was conducted at 300 W. The permeation of moieties through the SiO_4_-rich structure should be lower than through the crosslinked SiO_x_. This finding indicates that thickness variation (Figure 1a) is the main variable responsible for the increase in I_corr_ with an increase in P beyond 200 W in the curve of Figure 10b. To confirm this assumption, a new deposition procedure was conducted in plasma of 300 W, but increasing the deposition time to 1080 s. The resulting sample presented a thickness 3.6 times higher (1.1 µm) and a corrosion current 7 times lower (0.43 µA) than that prepared for 300 s (0.30 µm, 3.00 µA), showing the importance of barrier thickness to the final performance of the system.

Finally, Table 6 presents the corrosion current density (j_corr_) and corrosion potential (E_corr_) for the film prepared in this study at 300 W, along with its layer thickness. The corrosion current density was calculated using the formula j_corr_ = I_corr_/A, where A is the exposed surface area in the test (1.13 cm^2^). For comparison, the corresponding results for SiO_x_ films reported in the literature are also included in Table 6; all the data were obtained using 3.0–3.5% NaCl solution.

The current densities range from 10^−8^ to 10^−7^ A/cm^2^, while the corrosion potentials fall within the range of tenths of volts. Although the E_corr_ is similar across the different studies, the j_corr_ varies by up to 15 times. The thickness, ranging from 200 to 1400 nm (a 7-fold difference), cannot be the sole factor, as both the thickest (1400 nm) and thinnest (200 nm) layers provided the highest protection. Layers with intermediate thicknesses showed moderate protection, indicating that structural aspects also play a significant role. Nevertheless, the comparison confirms that the results obtained in this study fall within the range of values reported in the literature.

This set of results demonstrates the feasibility of using low-energy chemical reactions to create highly crosslinked or stochiometric silicon oxide structures via PECVD. No additional energy from ion bombardment or heating during or after deposition is required for this. The very same concept was previously employed by Rangel and co-workers to prepare highly crosslinked organosilicon films (SiO_x_C_y_H_z_) using HMDSO and Ar plasma mixtures [44]. In their study, the effects of plasma excitation power on the densification, structure, and mechanical and barrier properties of organosilicon films deposited on carbon steel were evaluated. The resulting films exhibited exceptional barrier and mechanical properties, since they were organosilicons. Considering all these findings, it is postulated here that this low-energy approach can also be extended to prepare other oxides (TiO_x_, AlO_x_) and carbides with unique structures from different metalorganic precursors. The idea is simply to tailor the removal of organics from the precursor molecules in order to properly promote crosslinking alongside oxidation.

## 4. Conclusions

Thin films were homogeneously deposited on the surface of different substrates from HMDSO, O_2_ and Ar plasma mixtures. The elevation of the power of the excitation signal promoted a trend of decrease in the deposition rate and film thickness, but did not change the overall chemical nature of the material. A silicon oxide network was created, with carbon detected only as a contaminant (≤1%). The proportions of silanol moieties and, consequently, of suboxide groups in the structure were reduced with increasing P. An amorphous tetrahedrical high-quality SiO_2_ matrix was created at the highest P level (300 W).

Film topography and roughness were dependent on deposition power. The transition from a uniform matrix to a granular surface and, eventually, to a fine-grained SiO_x_ smooth structure with increasing P corroborated the proposed modification in the deposition kinetics as a way of reducing C and OH inclusion and enhancing structural crosslinkings. The films showed good adherence to the substrates and did not crack or peel up under stress. The film hardness increased from 4.5 to 13.3 GPa when the power was enhanced from 100 to 300 W, reaching the hardness values of fused silica. The overall mechanical and tribological properties improved with increasing P, supporting the proposal of a better-connected structure with an increase in the energy delivered to the depositing plasma. After film deposition, the corrosion current was reduced by over 100 times compared to the untreated carbon steel, indicating a significant improvement in the corrosion resistance of this material. The degree of crosslinking and the quality of the SiO_x_ structure were the main factors influencing the mechanical properties, while the thickness of the barrier was also a relevant factor for corrosion resistance.

By means of these properties, it was possible to demonstrate that low-energy plasma phase and plasma surface reactions play the main roles in the reticulation of the structure and in its transformation into an almost complete stoichiometric network once ion bombardment is not effective. Therefore, it is possible to tailor the density of the SiO_x_ layer by controlling only the chemical reaction pathways.

In addition to confirming the tested hypothesis, the optimized deposition condition was identified. The film deposited under the 300 W condition improves both the corrosion and the scratching resistance of carbon steel, which will impact on the durability of the protection. The optimized process represents a single-step and low-energy method which does not require ion bombardment or material heating/annealing. Furthermore, the methodology is environmentally friendly, economically viable and scalable for large-scale production, offering potential for new industrial applications in areas such as corrosion protection, food and pharmaceutical packaging barriers.

## Figures and Tables

**Table 1:** Wavenumbers and assignments of bands detected in infrared spectra of films.

Wavenumbers (cm^−1^)	Vibrational Modes
445	Si-O-Si (rocking)
815	Si-O-Si (bending)
935	OH in Si-OH (stretching)
1062	Si-O-Si in SiO_x_ (stretching)
3500	OH in Si-OH (stretching)

**Table 2:** The atomic proportions of C, O and Si on the surface of the films deposited in plasmas of different excitation powers. The O/Si atomic ratios are also presented.

	Atomic Proportion			
Plasma Excitation Power (W)	C (%)	O (%)	Si (%)	O/Si
100	0.9	63.9	35.2	1.8
200	0.0	64.3	35.0	1.8
300	1.0	64.0	33.9	1.9

**Table 3:** Proportions of SiO_4_ (103.4 eV), SiO_2_ (102.0 eV) and Si-O (101.5 eV) groups in total area of high-resolution Si peak.

Plasma Excitation Power (W)	Si-O_4_ (103.4 eV)	Si-O_2_ (102.0 eV)	Si-O_1_ (101.5 eV)
100	92.3	7.7	-
200	91.6	8.4	-
300	98.5	-	1.5

**Table 4:** Hardness and reduced elastic modulus values for various SiO_x_ structures reported in the literature, along with those of the film deposited at 300 W in the present study.

Reference	Preparation Methodology	Deposition Parameter	Hardness (GPa)	Reduced Modulus (GPa)
Jung [67]	Thermal oxidation	1100 °C	11.5	72.5
Bartali [68]	Fused Silica	-	10.0	100.0
Puyo [70]	CVD	400 °C	7.2	62
Kasi [71]	CAP	150 W	0.55	10.5
Xu [72]	LP	600 W	16.4	118.9
Present work	LP	300 W	13.3	110.0
A.-Firouzjah [73]	LP	150 W	7.5	90–100
Jin [66]	LP	120 W	2.0	-

LP: low-pressure deposition; CAP: cold atmospheric plasma; CVD: chemical vapor deposition.

**Table 5:** The porosity of the films as a function of P.

P (W)	Porosity (%)
100	0.096
200	0.002
300	0.001

**Table 6:** The corrosion current density (j_corr_) and corrosion potential (E_corr_), together with the layer thickness for various SiO_x_ structures, reported in the literature and for the film deposited at 300 W in the present study.

Reference	Deposition Methodology	Deposition Power (W)	Deposition Temperature (°C)	Thickness (nm)	j_corr_ (μA/cm^2^)	E_corr_ (V)
Delimi, 2010 [78]	LP	10 W	300	200	0.0253	−0.66
Delimi, 2022 [79]	LP	10 W	300	400	0.27	−0.67
Present work	LP	300 W	No heating	1100	0.38	−0.62
Wang [80]	CAP–DBD	9 W	No heating	1400	0.024	−0.21

LP: low-pressure deposition; CAP: cold atmospheric plasma; DBD: dielectric barrier discharge.

## Data Availability

The original contributions presented in this study are included in the article. Further inquiries can be directed to the corresponding author.
